# Mesenchymal stem cell-loaded thermosensitive hydroxypropyl chitin hydrogel combined with a three-dimensional-printed poly(ε-caprolactone) /nano-hydroxyapatite scaffold to repair bone defects via osteogenesis, angiogenesis and immunomodulation

**DOI:** 10.7150/thno.39167

**Published:** 2020-01-01

**Authors:** Xiongfa Ji, Xi Yuan, Limin Ma, Bo Bi, Hao Zhu, Zehua Lei, Wenbin Liu, HongXu Pu, Jiawei Jiang, Xulin Jiang, Yu Zhang, Jun Xiao

**Affiliations:** 1 Department of Orthopedics, Guangdong General Hospital, Guangdong Academy of Medical Sciences, Guangzhou, Guangdong 510080, PR China.; 2 Department of Orthopaedic Surgery, Tongji Hospital, Tongji Medical College, Huazhong University of Science and Technology, Wuhan, 430030, China.; 3 Key Laboratory of Biomedical Polymers of Ministry of Education & Department of Chemistry, Wuhan University, Wuhan, 430072, China.

**Keywords:** thermosensitive hydrogel, 3D printed polymers, immunomodulation, angiogenesis, osteogenesis, osteointegration.

## Abstract

Chitin-derived hydrogels are commonly used in bone regeneration because of their high cell compatibility; however, their poor mechanical properties and little knowledge of the interaction between the materials and host cells have limited their practical application.

**Methods:** To evaluate osteoinductivity and enhance the mechanical properties of a newly synthesized thermosensitive hydroxypropyl chitin hydrogel (HPCH), a mesenchymal stem cell (MSC)-encapsulated HPCH was infused into a three-dimensional-printed poly (ε-caprolactone) (PCL)/ nano-hydroxyapatite (nHA) scaffold to form a hybrid scaffold. The mechanical properties and cell compatibility of the scaffold were tested. The interaction between macrophages and scaffold for angiogenesis and osteogenesis were explored *in vitro* and *in vivo*.

**Results:** The hybrid scaffold showed improved mechanical properties and high cell viability. When MSCs were encapsulated in HPCH, osteo-differentiation was promoted properly via endochondral ossification. The co-culture experiments showed that the hybrid scaffold facilitated growth factor secretion from macrophages, thus promoting vascularization and osteoinduction. The Transwell culture proved that MSCs modulated the inflammatory response of HPCH. Additionally, subcutaneous implantation of MSC-encapsulated HPCH confirmed M2 activation. *In situ* evaluation of calvarial defects confirmed that the repair was optimal in the MSC-loaded HPCH + PCL/nHA group.

**Conclusions:** PCL/nHA + HPCH hybrid scaffolds effectively promoted vascularization and osteoinduction via osteogenesis promotion and immunomodulation, which suggests promising applications for bone regeneration.

## Introduction

There is an increasing demand for large bone grafts to heal massive bone defects in patients after revision surgery, malignancy resection, infection, or severe trauma 1. Currently, autologous bone grafts are widely used to repair bone defects in the abovementioned diseases; however, their limited availability and the risk of injury with additional surgery has restricted their clinical application 2. Additionally, these autologous bone grafts cannot adapt precisely to the complicated shape of the defect after surgery. Bone tissue engineering (BTE) is an alternative solution that uses scaffolds of natural or synthetic biomaterials to promote bone regeneration 3, 4. The conventional BTE fabrication process uses solvent casting, gas forming, or freezing and can only produce randomly-formed internal structures inside the scaffold 5. Alternatively, three-dimensional (3D) printing, a cutting-edge technique, is used to fabricate scaffolds with customizable shapes and inner structures 6. The combination of 3D printing and BTE shows promise and has been extensively studied over the past several decades 7.

Currently, extrusion-based printing is the most widely used and cost-effective technique to fabricate 3D interconnected porous scaffolds 8. Synthetized polymers have good printability and high mechanical strength; however, their high melting points and viscous states have a negative influence on the activity, and even the distribution, of bioactive compounds. Low-temperature printing is a promising method to print drug-loaded biomaterials for tissue engineering 9. Poly (ε-caprolactone) (PCL) is an FDA-approved polymer with biodegradable properties, nontoxic degradable byproducts, and good compatibility with 3D printing 10. Thus, PCL is suitable for low-temperature 3D printing of BTE. However, the hydrophobicity of PCL, and its lack of surface cell recognition sites 11, has limited its use in BTE. It has been reported that hydroxyapatite (HA) particles can stimulate macrophages to secrete angiogenic 12 and osteogenic 13 growth factors. Vascularization and osteoinduction are two important processes for bone regeneration; thus, it is reasonable to incorporate HA into 3D-printed PCL scaffolds.

Compared to synthetic materials, natural materials, such as hydrogels, show optimal cell compatibility and high nutrient content and thus are suitable for cell encapsulation and bioactive drug delivery 14, 15. Thermosensitive hydrogels have good mobility at relatively low temperatures and can convert to a gel state at normal physiological temperatures *in vivo* without toxic additives. Therefore, cells and bioactive compounds can easily be homogeneously distributed in a solution state, and *in situ* administration with fast sol-gel transitions below body temperature is convenient. Biodegradable thermosensitive hydrogels that produce nontoxic byproducts will provide further benefits for *in vivo* applications where degradation is desired. Due to these advantages, thermosensitive hydrogel is regarded as a promising delivery platform in tissue engineering 16. In our previous work, we homogeneously synthesized a new thermosensitive hydroxypropyl chitin hydrogel (HPCH) using a “green” method 17; thus, HPCH demonstrated good biocompatibility and biodegradability. Importantly, the fabrication process is stable, reproducible, and inexpensive. Sensitive sol-gel transformation is useful for *in situ* injection after loading with cells or drugs. However, similar to other biodegradable hydrogels, it is too weak to retain its shape, especially when the defects are large. Therefore, it is reasonable to combine synthetic polymer PCL and natural material-based hydrogel HPCH to create a mixed scaffold with high cell compatibility and good mechanical properties 18.

Implantation of biomaterials may induce inflammation and local tissue injury, through activation of macrophages 19, 20. Thus, the interaction between implanted biomaterials and immune response should be considered. It is important to regulate the immune response toward homeostasis as opposed to chronic inflammation 21. Mesenchymal stem cells (MSCs) are a key component in tissue engineering, as they have the ability to differentiate into bone, cartilage, and marrow adipocytes 22, 23. Studies have shown that MSCs have low immunogenicity, immune-masking properties, and immunomodulatory capabilities 24. More importantly, MSCs can promote macrophage transition from classically activated (M1) to alternatively activated (M2) through paracrine mechanisms, producing cytokines for anti-inflammatories and tissue regeneration 25. However, as discussed previously, an inflammatory environment may be harmful to MSCs 26. When implanted *in vivo*, the survival rate of MSCs seeded on a printed scaffold is low, and the promotion of bone healing is limited 27. As mentioned earlier, hydrogels were shown to be good cell carriers in various *in vivo* studies 28, 29. The hydrogel effectively acts as a “protector” of MSCs^30^; additionally, the trophic factors released by MSCs may attenuate the foreign body response of HPCH and regulate macrophage transition toward M2.

In our research, we examined the efficiency of HPCH for MSC delivery and the immune system regulation that occurs when a hydrogel is introduced to a 3D-printed scaffold. HA was used in this hybrid scaffold to direct MSC bone differentiation. We hypothesized that HPCH effectively encapsulates MSCs, and that the hybrid scaffold regulates macrophage transition to M2, which may enhance bone healing.

## Methods

### Preparation of HPCH/MSCs + PCL/nHA scaffold

**Figure 1:**
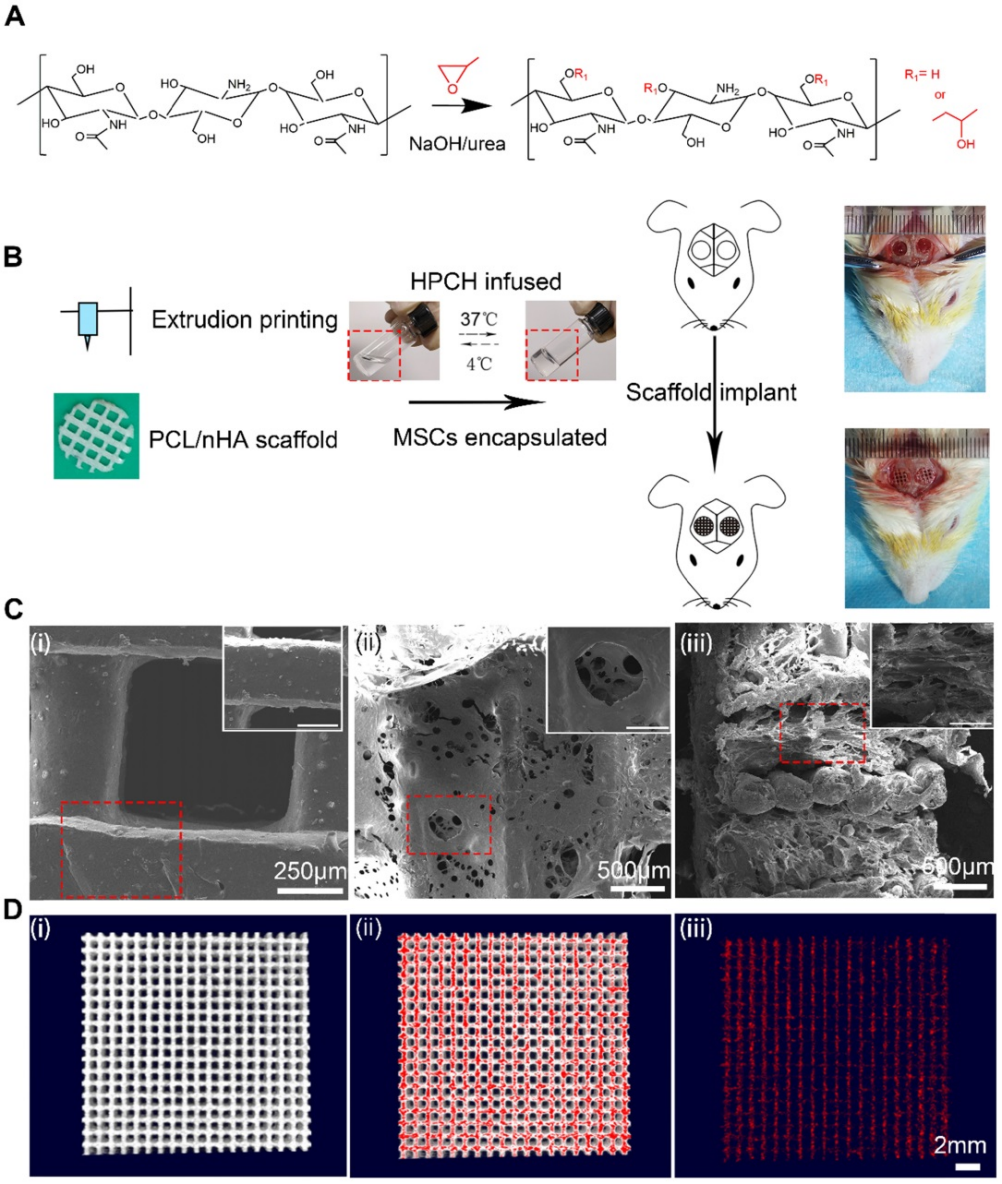
** The characterization of PCL/nHA+HPCH scaffold.** (A) the synthesis process of HPCH. (B)The schema of Hybrid scaffold fabrication and *in vivo* implant. (C) SEM of the PCL/nHA+HPCH scaffold. (i) Micro-structure of PCL/nHA scaffold and nHA distribution in the scaffold. (ii) Front view of micro-structure of PCL/nHA+HPCH scaffold. (iii) Side view of micro-structure of PCL/nHA+HPCH scaffold. The scale bar in the magnification window was 200 μm. (D) The micro-CT reconstruction of PCL/nHA. The 3D structure of PCL/nHA was showed in (i), and red color was used to label the high contrast nHA (ii). PCL was subtracted and the remained nHA showed in (iii). Scale bar, 2 mm.

PCL (number-average molecular weight = 84,200 Da) and nano-hydroxyapatite (nHA) were purchased from Sigma-Aldrich (USA). The scanning electron microscopy (SEM) characterization of nHA is shown in Fig. S1. To prepare suspensions for the 3D printing of polymer-hydroxyapatite composite scaffolds, CH_2_Cl_2_ was used to dissolve PCL, and nHA was gently stirred into the PCL solution using a homogenizer 31. A composite 3D printed scaffold with 30% nHA is endowed with a biomimetic structural and chemical composition similar to that of native bones 32, thus we chose a PCL: nHA ratio of 2:1. While stirring, the suspension was heated to boil to evaporate the solvent and achieve a suitable viscosity for the printing. To produce the scaffold, an extruding 3D printer (Hangzhou Regenovo Biotechnology, China) with a 0.4-mm diameter needle was used. The speed of the nozzle was set at 4 mm·s^-1^. The strands with 1 mm spacing were dispensed layer by layer, forming 0°- to 90°-orientated junctions. With a Z axis interlayer increment of 0.2 mm, five layers in each scaffold were fabricated to fit the calvarial thickness. After printing, all the scaffolds were kept at room temperature to evaporate the residual solvent. Cylindrical disks were punched out from the scaffolds using a 4-mm diameter corneal trephine. The fabrication procedure is shown in Fig. 1B. All scaffolds were sterilized using 75% ethanol with UV light for 48 h and then rinsed twice with PBS for further use.

HPCH was homogeneously prepared in NaOH/urea solution according to our previous study 33. Briefly, chitin was dissolved in NaOH/urea solution and reacted with propylene oxide under vigorous stirring at 15°C for 24 h (Fig. 1A). After it had been neutralized, the solution was dialyzed and freeze dried. HPCH was dissolved in PBS to prepare 2 wt% solution for further use.

To fabricate HPCH/MSCs + PCL/nHA hybrid scaffolds, we first blended 10^6^/mL of the cells with the HPCH precursor solution at 4 ℃ for 5 min to form an HPCH precursor mixture. Subsequently, the precooled HPCH mixture was incorporated with PCL/nHA in a 1 mL syringe with negative pressure to ensure that the hydrogel precursor solution fully filled the scaffold. Finally, the syringe was incubated at 37 ℃ for 30 min to form the gel, and it was then removed from the tube for further use.

### Characterization of HPCH + PCL/nHA scaffold

SEM (Quanta 250, FEI, USA) was used to investigate pore morphology of the porous PCL/nHA and HPCH + PCL/nHA scaffold, according to a previous report 34.

The mechanical properties were determined by unconfined compression tests. The scaffolds were pre-loaded with a force of 0.05 N (N = 4). The stress-strain curve of the scaffold specimens was recorded using a universal testing machine (Instron 5565, USA) at a constant loading rate of 1 mm /min, and the compressive modulus was calculated based on the slope of the curve.

To evaluate the distribution of nHA in PCL, a Scanco Medical 40 Micro-CT system (Scanco Medical, Bassersdorf, Switzerland) with an X-ray source of 70 kVP and 114 μA was used to scan all samples using the 20 mm x 20 mm printed PCL/nHA scaffold as a scanning sample. The 3D reconstruction of the PCL/nHA scaffold was carried out using Skyscan CTvox 2.1 software. The high density nHA was isotopically labeled using red color, and the 3D structure was shown in a video (Video 1).

### Cell compatibility of HPCH + PCL/nHA

RAW264.7, stored in our lab, was cultured in α-MEM (Hyclone, USA) supplemented with 10% fetal bovine serum (FBS), penicillin (100 U/ml), and streptomycin (100 mM/ml) at 37 ℃ in a 5% CO_2_ atmosphere. MSCs were extracted from four-week-old Sprague-Dawley (SD) rats according to previous reports 35 and cultured in DMEM supplemented with 10% FBS, penicillin (100 U/ml), and streptomycin (100 mM/ml) at 37 ℃ in a 5% CO_2_ atmosphere. Passage 3 of MSCs was used for the following experiments. Rat endothelial cells were isolated from rat aorta according to a previously published article 36. CD31 fluorescent staining was used to identify the endothelium cells (ECs) (Fig. S2).

The cell viability of MSCs encapsulated in the hydrogels after seven days' culture was evaluated by a Live/Dead Viability/Cytotoxicity Assay Kit (Invitrogen) using confocal laser scanning microscopy (CLSM, Olympus FV 1000, USA). The cytotoxicity of HPCH + PCL/nHA was evaluated in MSCs by CCK-8 assay. The cells were seeded on the bottom of 96-well plates with a density of 5,000 cells/well. Condition culture medium at 100 µL from HPCH, PCL/nHA, and HPCH + PCL/nHA was added, while the HPCH-less culture medium was used as control. The medium changed every day. After incubation for 24, 48, and 96 h, CCK8 was used to test the cell viability. Flow cytometry was performed to measure the influence of HPCH on the apoptosis rate of MSCs. Shortly after, HPCH was added at 3 mg/mL or 6 mg/mL, and blank plated-MSCs were used as the negative control. After 24 h, cells were harvested, washed, and stained with FITC-Annexin V and propidium iodide (PI) (BD Biosciences, USA) and subsequently analyzed using a flow cytometer (BD Biosciences, USA).

To evaluate cell adhesion on HPCH, hydrogels (60 µL, 2% w/v) were formed in 96-well plates at 37 ℃. Subsequently, MSCs and RAW were seeded on the surfaces of HPCH or 96-well plates (as control) at a density of 5,000 cells/ well, and incubated at 37°C in a 5% CO_2_ atmosphere. One day after culturing, the cells were stained by FITC labeled phalloidin and 4', 6-diamidino-2-phenylindole (DAPI) to visualize their cellular morphology, and were viewed by a fluorescence microscope (Evos FL Auto, USA).

### RT-PCR test

The total RNA was extracted using an E.Z.N.A.®Total RNA Kit I (Omega, USA), according to the manufacturer's instructions. Subsequently, the mRNA was converted to complementary DNA (cDNA) using a ReverTra Ace® qPCR RT Kit (Toyobo, Japan). The RT-PCR was carried out using a CFX Connect Real-Time PCR Detection System (BIO-RAD, USA). The relative amount of gene transcripts was normalized to GAPDH. The primers are listed in Table S1 and Table S2.

### Alkaline phosphatase (ALP) fluorescent staining

For the visualization of ALP expression, MSCs were incubated with rabbit anti-rat ALP (1:100, Abacam) followed by Alexa Fluor488 Conjugate anti-rabbit IgG secondary antibody (1:1000, Cell Signaling Technology) for ALP staining. The images were acquired using the CLSM (Olympus FV1000, Japan) and processed with Image-J.

### Matrigel assay

A tubule-forming assay was used to assess the ability of angiogenesis growth factors that had been released from the macrophages cultured with HPCH/nHA scaffold to promote vascularization. Matrigel™ was briefly placed in a 96-well plate (60 μL/well) and incubated for 30 min. Rat ECs were then inoculated at a density of 10^4^ cells/well with 100 μL of conditional culture medium (Fig. 4A). Matrigel cultures were imaged at 8 h with a Nikon microscope (10× objective). For each group, six images were taken, and they were analyzed using Image J software (Angiogenesis package). The number of segments, number of nodes, and length of the tubules were used as quantitative analysis of angiogenesis.

### Transwell culture

The Transwell plate was used to evaluate the immunomodulation of MSCs-HPCH on macrophages. In this case, 10^5^ RAW264.7 cells were cultured in the lower chamber, and LPS (10 ng/mL) was used to stimulate RAW264.7 cells from M0 to M1. In the upper chamber, 10^5^ MSCs were cultured. HPCH was diluted to 3 mg/mL using normal culture medium (Fig. 6A). After co-culturing for 6 h, the total mRNA of the RAW264.7 cells was collected and analyzed using qRT-PCR.

### Subcutaneous implantation

To evaluate the efficiency of MSC-loaded HPCH to modulate the phenotype of macrophages, HPCH with and without MSCs was transplanted into subcutaneous pockets and harvested after 1, 4, and 7 days of implantation for immunofluorescence analyzes. Samples were fixed with 4% paraformaldehyde for 48 h, embedded in paraffin, and sectioned into 4-μm sections. To assess the M1 and M2 macrophages surrounding the hydrogel, sections were immunohistochemically stained using primary antibodies specific for CD206 (Proteintech, USA) and iNOS (Proteintech, USA). Alexa Fluor 488 and 594 (Proteintech, USA) were applied appropriately. The cell nuclei were counter stained with DAPI, and the images were analyzed using Image J software.

### Establishment of rat calvarial defect model

All animal experimental procedures were approved by the Animal Care and Use Committee, the Huazhong University of Science and Technology. Male SD rats (age = 3 months; weight = 300-350 g) were maintained in the animal facility for one week to acclimatize the diet, water, and housing conditions. The animals were divided into three groups (five rats/group): defect only (control); PCL/nHA; PCL/nHA with HPCH (Fig. 1B). Prior to operation, the animals were anesthetized with pentobarbital sodium (1%, 50 mg/kg) by intraperitoneal injection. The surgical site was disinfected with iodine. Using sterile instruments and aseptic technique, a 2-cm cranial skin incision centered over the sagittal suture was made down to the periosteum. The periosteum was divided in line with the skin incision and elevated as a single flap. Two 4-mm diameter, bicortical, extra-dural defects were created over both sides of the parietal bone using a dental trephine drill, and precooled saline was used to prevent overheating of the bone margins 37. The periosteal flap functioned as an envelope to contain the implants (Fig. 1A). The periosteum and skin were repositioned and closed using 4-0 sutures 38. SD rats were sacrificed after nine weeks, and the whole calvaria, along with surrounding bone and soft tissue samples, were harvested for subsequent micro-CT and histological analyzes. All samples were fixed in 4% paraformaldehyde for three days before decalcification.

### Microcomputed tomography (micro-CT) analysis

Micro CT was performed on all samples using a Scanco Medical 40 Micro-CT system (µCT40; Scanco Medical, Bassersdorf, Switzerland), with an X-ray source of 70 kVP and 114 μA. The defective region along with the surrounding bone was reconstructed, and a cylindrical volume of interest (VOI;4 mm radius, 0.8 mm deep) was defined to assess bone healing using Mimics Medical 19.0 (Materialise Corp., Leuven, Belgium). Repair was expressed as bone volume.

### Histological and immunohistochemical analysis

Following micro-CT, the specimens were decalcified in 10% ethylenediaminetetraacetic acid (EDTA) solution for eight weeks, dehydrated in a series of graded ethanols, and embedded in paraffin. Decalcified specimens were then sectioned into 4-μm slices from the vertical to the sagittal suture across the center of each calvarial defect using a microtome (LEICA, SM2000R). Hematoxylin and eosin (H&E) and Masson's trichrome and CD31 staining were used for histological and histomorphometric analyses. Digital images of the stained sections were obtained using the Evos FL Auto microscope.

### Statistical analysis

All data are presented as mean ± standard deviation (SD). All experiments were performed in at least three replicates. Statistical analysis was performed using a two-way analysis of variance test, followed by Tukey's honestly significant difference post-hoc test to evaluate differences between treatment groups, and a multiple *t-*test, followed by the Holm-Sidak method, to evaluate differences in each group. The level of significance was set at *p <* 0.05. All analyzes were carried out using GraphPad Prism 6.0 (GraphPad Software, San Diego, CA, USA).

## Results

### Characterization of 3D-printed PCL/nano-hydroxyapatite (nHA) scaffold and HPCH-infused hybrid scaffold

We first aimed to establish a method for 3D printing of a scaffold with good cell compatibility and mechanical properties. To this end, a mixture of PCL/nHA (2:1) was 3D printed onto a glass slide using an extrusion printer. MSCs were encapsulated in HPCH at 4℃ under flow conditions and then infused into the printed scaffold to form the PCL/nHA + HPCH hybrid scaffold (Fig. 1B), which was allowed to stabilize at 37℃ for 30 min. The distance between two strands was set at 1 mm; however, the actual size of the hole was about 800 μm (Fig. 1C). The freeze dried hybrid scaffold showed interconnected micro-channels of 50-200 μm (Fig. 1C), which were beneficial for imparting nutrition and for cell connection 39. SEM images revealed that the HPCH had fully infiltrated the PCL/nHA scaffold (Fig. 1C). Micro-CT showed nHA distributed throughout PCL (Fig. 1D, Video 1). The Young's modulus of the printed PCL/nHA structures under compression was 2.34 ± 0.10 MPa (N = 4) and 2.33 ± 0.27 MPa (N = 4) for the hybrid constructs, which was much higher than that of the HPCH hydrogel (1.0 kPa) 17.

### Cell compatibility of the HPCH + PCL/nHA scaffold

**Figure 2:**
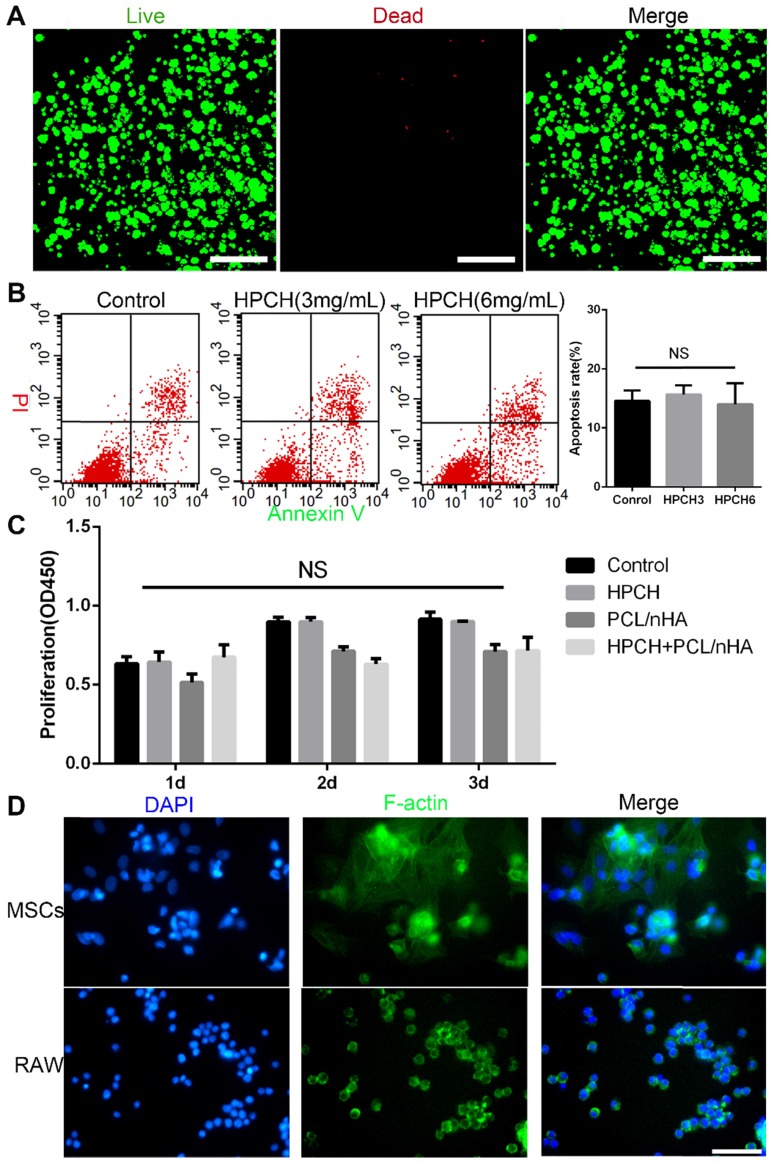
** Cell viability and adhesion of PCL/nHA+HPCH scaffold.** (A) Live/Dead assay of MSCs cultured in HPCH after 7 days. (B) Apoptosis rate of HPCH-treated apoptotic MSCs for one-day culture. (C) The CCK8 assay of MSCs when cultured with PCL/nHA and HPCH after 1d, 2d and 3d. (D) Phalloidin staining of MSCs and RAW264.7 cultured on HPCH for 1 day. Scale bar, 100 μm. NS, not significant (P < 0.05).

Live/Dead staining showed that the HPCH hydrogel was nontoxic to the encapsulated MSCs after seven-day culture (Fig. 2A). The Cell Counting kit-8 (CCK8) (Dojindo, Kumamoto, Japan) indicated no significant differences in proliferation among the control, HPCH, PCL/nHA, and HPCH + PCL/nHA groups on days 1, 2, or 3. Apoptosis test results showed that HPCH had no influence on the apoptosis rate of MSCs.

According to our previous research on cell adhesion, cells such as Hela cells tend to aggregate on the surface of the HPCH 40. Thus, we tested the cell adhesion of different cell types on HPCH. Our results indicated that all types of cells tended to form a colony on the surface (Fig. 2D) (Fig. S3). Although MSCs also aggregated, the cells spread over the hydrogel, which meant that MSCs communicated with hydrogel in a 3D environment (Fig. 2D). RAW 264.7 cells retained their round shape without membrane spreading or filopodia, which indicated that GM-HPCH did not stimulate significant inflammatory response (Fig. 2D).

### Osteogenesis of the HPCH + PCL/nHA scaffold

**Figure 3:**
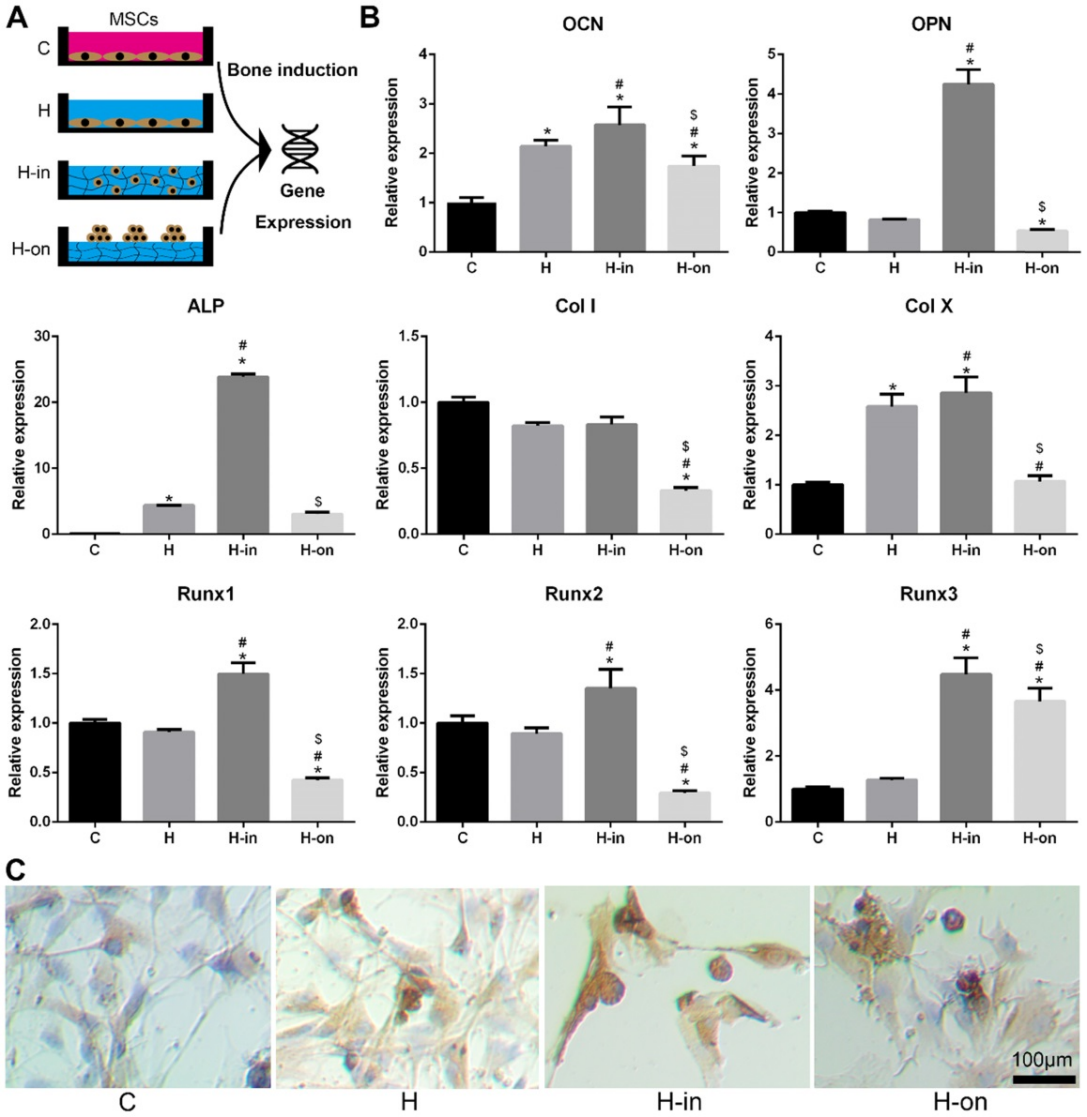
** Spatial influence of HPCH on MSC osteogenesis.** (A) The schematic illustration of cultured modes of MSCs on HPCH. (B) RT-PCR of osteogenic genes. (C) OCN immunochemistry staining of MSCs cultured in or cultured on HPCH. Scale bar, 100 μm.

When MSCs were cultured with HPCH, we found that HPCH spatially influenced MSC behavior (Fig. S2). MSCs tended to aggregate on the HPCH surface and retained their round shape when encapsulated in HPCH. The MSCs seeded on the culture plate, with or without HPCH solution (3 mg/mL), were used as the control (Fig. 3A). RT-PCR results showed that when MSCs were encapsulated in HPCH (H-in), the gene expression levels of osteocalcin (OCN) and osteopontin (OPN), which are related to osteogenesis, increased significantly. Runx1 is known to be associated with chondrogenesis 41, whereas Runx2 and Runx3 are related to endochondral bone formation 42. In our H-in group, the gene expression levels of Runx1, Runx2, and Runx3 all increased significantly; thus, osteogenesis in the H-in group may have been related to the endochondral process. The increased expression of Col X, a gene associated with chondrocyte hypertrophy, supports this proposal. Interestingly, in the H-on group, all bone-related genes were suppressed, especially Col I, indicating that the aggregation of MSCs on the HPCH surface benefited chondrogenesis, but not osteogenesis (Fig. 3B). Immunochemistry staining of OCN was consistent with the PCR results (Fig. 3C); MSC encapsulation in HPCH resulted in greater OCN expression.

**Figure 4:**
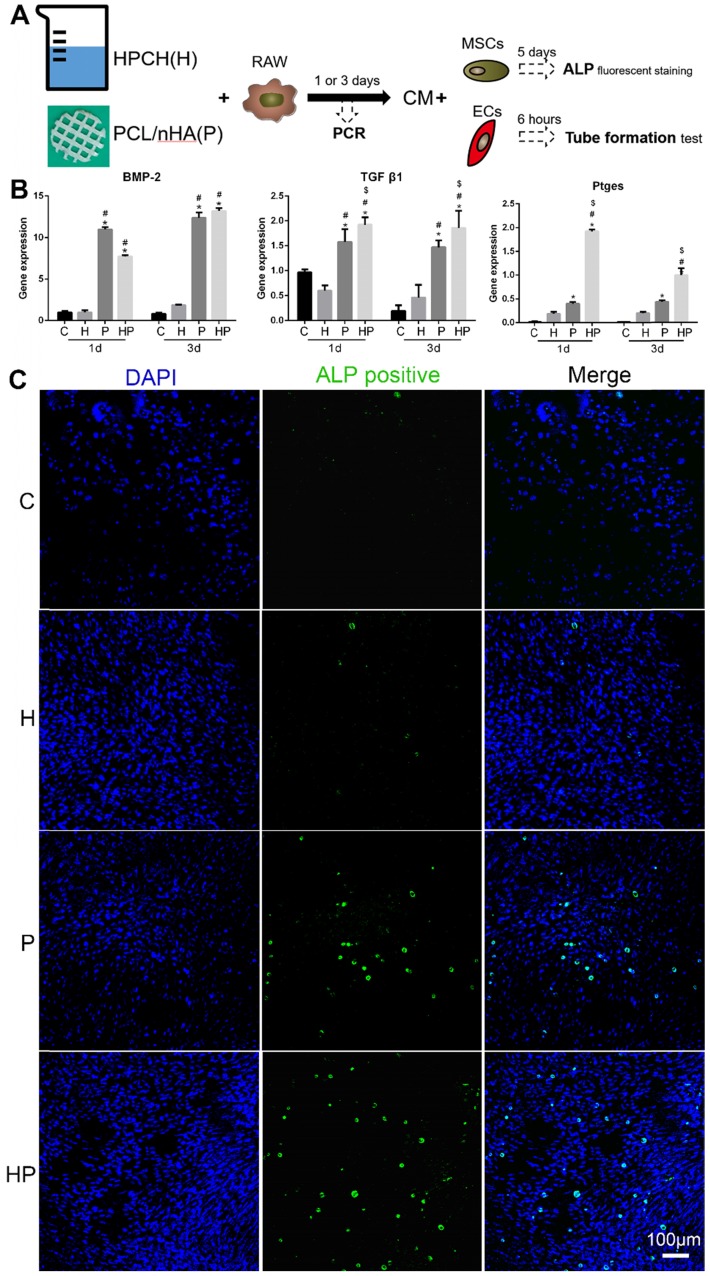
** Osteoinduction of PCL/nHA+HPCH via macrophage secretion.** (A) The schematic illustration of osteogenesis and angiogenesis of the extract medium from RAW 264.7, which was cultured with PCL/nHA and HPCH for one day or three days. (B) Osteo-induced growth factor related gene expression of RAW 264.7 treated with PCL/nHA and HPCH. (C) ALP fluorescent staining of MSCs treated with extract medium from RAW 264.7 cultured with PCL/nHA and HPCH. C, control; H, HPCH; P, PCL/nHA; HP, HPCH+PCL/nHA. *P < 0.05 versus Control; #P < 0.05 versus HPCH; $P < 0.05 versus PCL/nHA.

To further investigate the beneficial effects of the HPCH + PCL/nHA scaffold on bone formation, it was cultured with macrophages. Interestingly, the gene expression levels of bone morphogenetic protein-2 (BMP2), transforming growth factor-beta 1 (TGF-β1), and prostaglandin E2 (PGE2), all of which are growth factors related to bone formation, were increased in the HPCH + PCL/nHA group (Fig. 4B). To assess the functional role of macrophage-secreted factors in osteogenesis, ALP staining was performed. Consistent with the PCR results, a larger number of ALP-positive cells were observed in the HPCH + PCL/nHA group (Fig.4C), and more calcium nodes were found in the PCL/nHA group using Alizarin red staining (Fig. S4); this indicated that the HPCH + PCL/nHA scaffold can initiate bone formation through macrophage activation.

### Angiogenesis of the HPCH + PCL/nHA scaffold via macrophage activation

**Figure 5:**
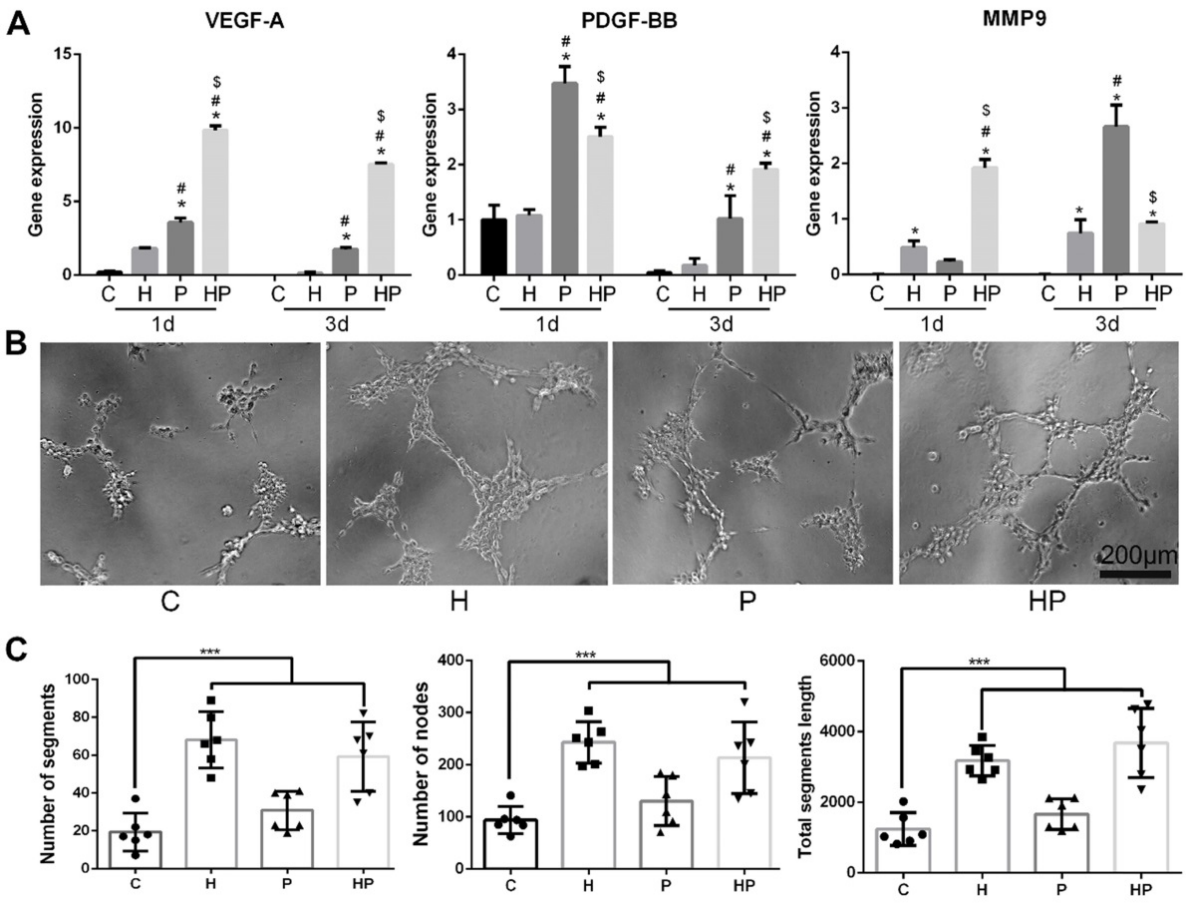
** Angiogenesis of PCL/nHA+HPCH via macrophage secretion.** (A) Angiogenesis related gene expression of RAW 264.7 treated with PCL/nHA and HPCH. (B) The bright-field images of tubule formation of ECs treated with extract medium from RAW 264.7 when cultured with PCL/nHA and HPCH. (C) Quantitation of tubule formation results.

**Figure 6:**
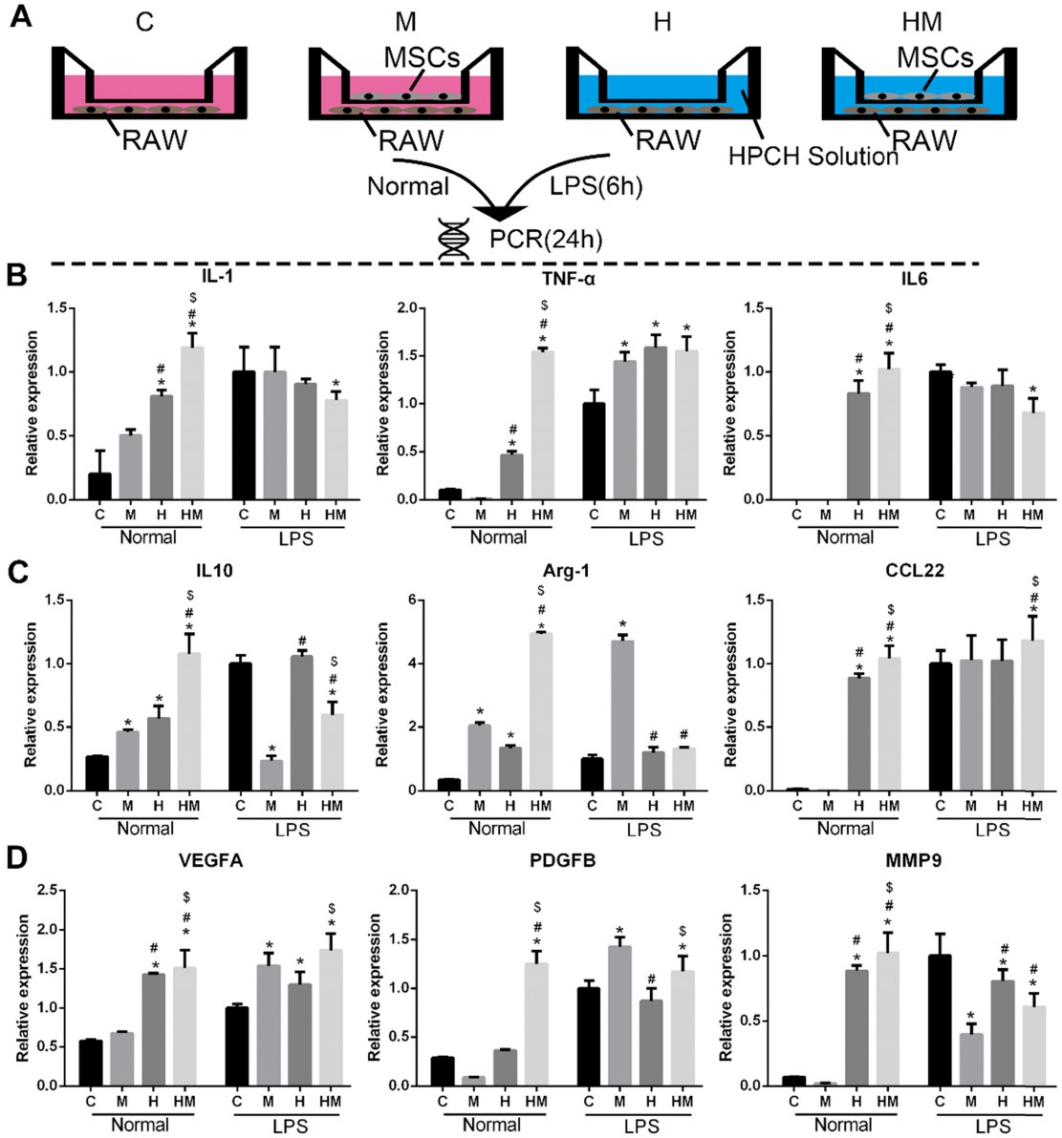
** Immunomodulatory effect of MSCs-HPCH on macrophages under normal condition or LPS stimulation.** (A) The schema of Transwell culture of MSCs and RAWs with HPCH hydrogel. (B) M1 related gene expression; (C) M2 related gene expression; (D) Angiogenesis related gene expression. C, control; M, MSCs; H, HPCH; HM, HPCH-MSCs. *P < 0.05 versus Control; #P < 0.05 versus MSCs; $P < 0.05 versus HPCH.

We subsequently analyzed the expression levels of genes involved in angiogenesis. Vascular endothelial growth factor (VEGF), a gene involved in the initiation of angiogenesis, increased in macrophages when cultured with PCL/nHA; the inclusion of HPCH can significantly amplify this effect, as shown in Figs. 5A and 6D. Thus, these results suggest that the composite scaffold promotes macrophages to secrete VEGF and thus initiate angiogenesis. Platelet-derived growth factor-BB (PDGF-BB), which is known to recruit pericytes and stabilize the vasculature 43, increased in macrophages cultured with PCL/nHA (Fig. 5A). Although HPCH had no significant influence on PDGF-BB expression, the presence of MSCs and HPCH can result in the expression of high levels of PDGF-BB (Fig. 6D). Therefore, both PCL/nHA and MSCs may help to stabilize angiogenesis by activating macrophages to secrete PDGF-BB. Matrix metalloproteinase-9 (MMP-9), which contributes to the remodeling of the extracellular matrix and thus to allowing ECs to migrate, partly stimulated angiogenesis 43. We found that HPCH can promote MMP-9 expression in the early stage (1 day), and PCL/nHA gradually promotes MMP-9 gene expression (three days), consistent with the degradation rates of HPCH and PCL/nHA.

To assess the effects of interaction between macrophages and biomaterials on angiogenesis, a tube formation assay was performed. More segments, nodes, and length of the tubules of networks of ECs were observed in conditioned media, including HPCH compared with the control and PCL/nHA groups. Interestingly, conditioned media from the PCL/nHA group also exhibited increased sprouting compared with the control group (Fig. 5B and C). Overall, PCL/nHA + HPCH had a positive effect on angiogenesis via macrophage activation.

### Immunomodulatory effects of MSCs-HPCH on macrophages

To verify the immunomodulatory effects of MSCs on HPCH-induced macrophage activation, a Transwell test was conducted, in which MSCs and RAW264.7 cells were cultured in the upper chamber and the lower chamber, respectively. RAW264.7 was pre-stimulated with lipopolysaccharide (LPS) for 6 h; RAW264.7 cells not stimulated by LPS were used as the control. The RT-PCR results showed that, under normal conditions, HPCH can promote both M1 (interleukin-1 [IL-1], tumor necrosis factor-alpha [TNF-α], and IL-6) and M2 (IL-10, arginase-1 [Arg-1], and C-C motif chemokine ligand-22 [CCL22]) gene expression. MSCs can amplify these effects; however, with LPS stimulation, MSCs not only reduce M1-related gene expression, but also promote M2-related gene expression (Fig. 6). Thus, in an inflammatory environment, MSCs can suppress HPCH-induced M1-related gene expression and promote macrophage transition toward M2, thus initiating tissue regeneration.

**Figure 7:**
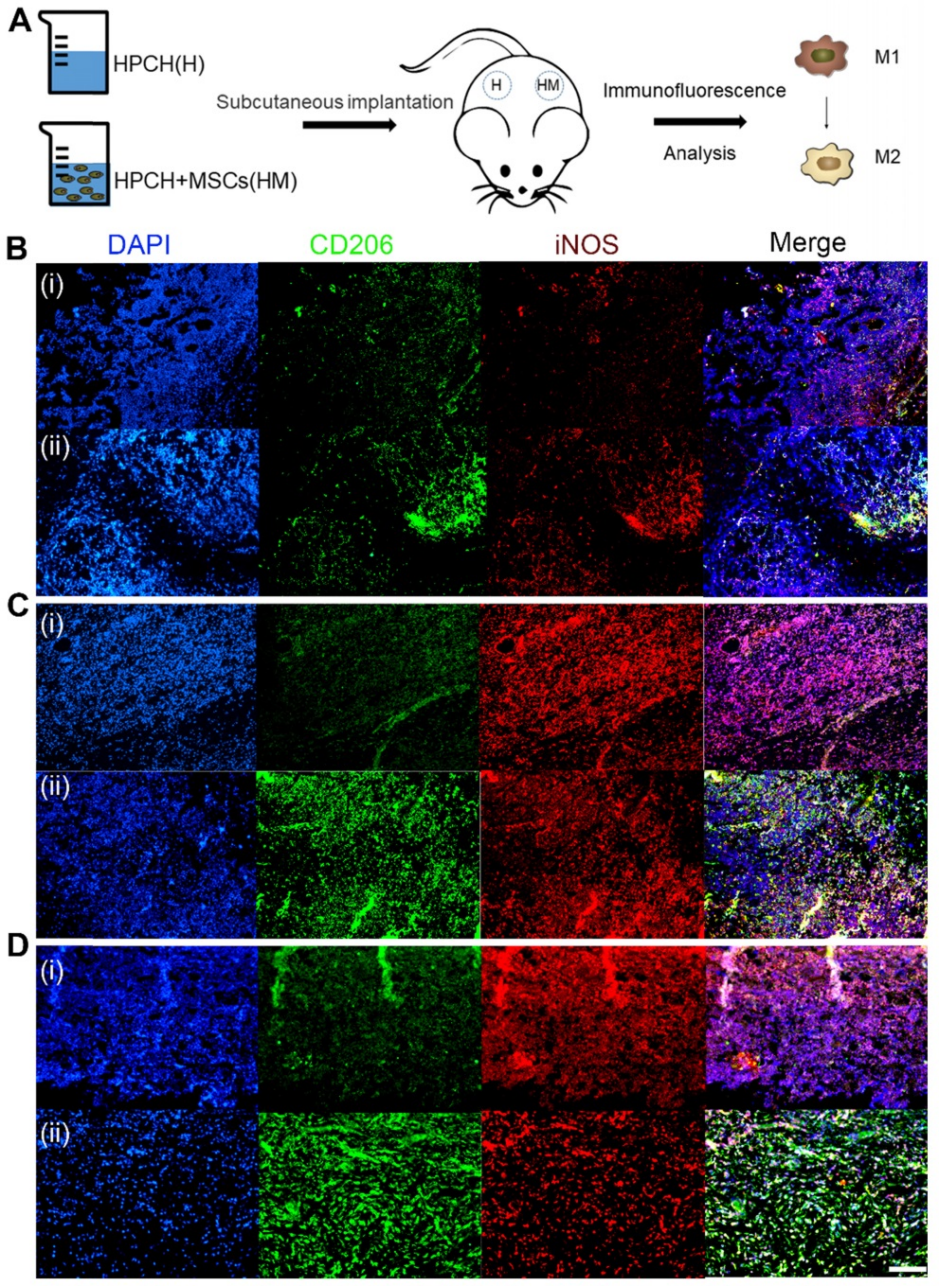
** Immunomodulation of MSCs-HPCH on macrophages *in vivo*.** (A) The schema of subcutaneous implantation of HPCH and MSCs-HPCH hydrogel. (B) Immunofluorescent staining of subcutaneous implanted hydrogel in 1 day (B), 4 days (C) and 7 days (D). iNOS was used to label M1 macrophages, and CD 206 was used to label M2 macrophages. The implants at each time point were HPCH (a) and MSCs-HPCH (b). Scale bar, 100 μm.

To further verify the *in vitro* results, immunofluorescence-labeled staining was performed on the macrophages surrounding the subcutaneous HPCH and MSCs-HPCH implants. And the MSC injection group was used as control (Fig. S5). The H&E staining showed that MSCs caused low level of local inflammatory response, but the HPCH caused extensive inflammatory response, which was the main reason for the local lymphocyte infiltration (Fig. S6). On the first day of implantation, the inflammatory cells started to infiltrate into the hydrogel, and the majority were iNOS-positive macrophages. On day 4, the number of inflammatory cells had increased significantly, and the number of CD206 positive cells in the MSCs-HPCH group eventually exceeded that of iNOS-positive macrophages, while iNOS-positive cells were still in the majority in the HPCH group. Thus, MSCs had an immune-regulating effect on the surrounding macrophages and promoted their transition to the M2 state. On day 7, inflammatory cells remained in the HPCH group (mainly iNOS-positive cells), whereas there were significantly fewer inflammatory cells in the MSCs-HPCH group (mainly CD206-positive cells). This indicates that MSCs can promote the transition from an inflammatory state to a regenerative state (Fig. 7).

### Bone repair using MSCs-HPCH + PCL/nHA hybrid scaffold

**Figure 8:**
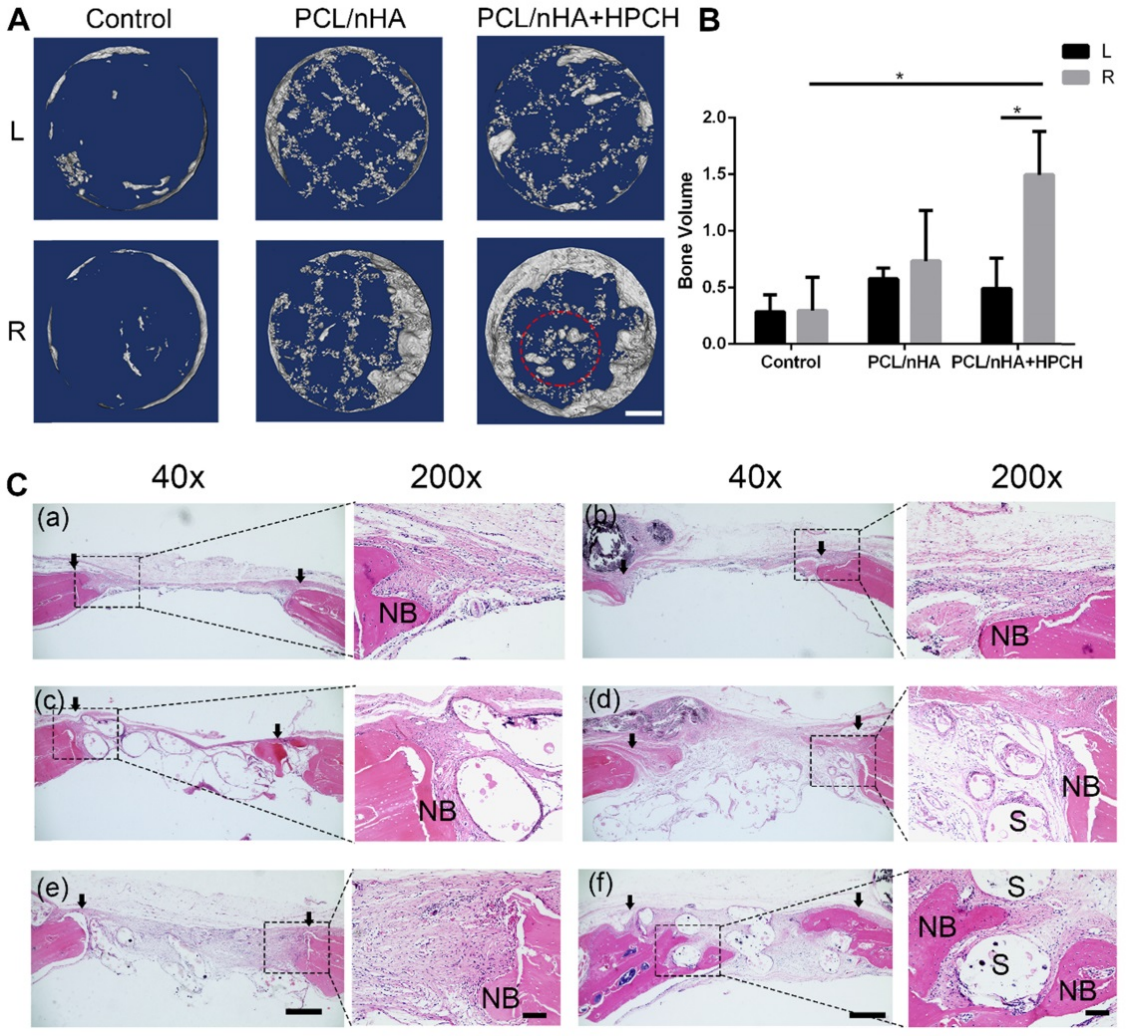
** Micro-CT and H&E histologic evaluation of *in vivo* calvarial defect repair at 9 weeks of post-implantation.** (A) The representative micro-CT images of the control, PCL/nHA, PCL/nHA+HPCH group in the left and right defect. Red dotted circle indicates spheroid bone formation in the central of scaffold. (B) Quantitative result of bone volume in different groups. * represents p<0.05. Scale bar, 1mm. (C) The representative whole and local photomicrographs of HE staining images of calvarial defects at 9 weeks of post-surgery. The newly formed bone in control group (a-b), PCL/nHA group (c-d), PCL/nHA+HPCH group (e-f), where (a), (c), (e) were the left defect and (b), (d), (f) were the right defect. 200X image in each group was the magnification of newly formed bone either in margin or in central of the defect. NB, new bone; S, scaffold. The arrows indicate the boundary of bone defect and native bone. Scale bar: 500 μm in 40X, and 100 μm in 200X.

Micro-CT analysis was used to visualize and quantify bone formation within the defects at nine weeks after implantation. New bone growth was observed in the margins of the defects in all groups, which indicates good osteoconduction of the PCL/nHA scaffold. Over the nine-week period, a significant difference emerged in the right-sided defect of the PCL/nHA + HPCH group, compared with the PCL/nHA and control groups with MSCs (*p* < 0.05). No significant difference in total bone volume was found in the MSC-free group (left defect, *p* > 0.05). However, in the PCL/nHA + HPCH group, the hybrid scaffold encapsulated with MSCs (right side) exhibited a significant (three-fold) increase in new bone volume compared with the scaffold without cells (left side, *p* < 0.05). However, no significant difference was found in the presence of MSCs between the other groups (Fig. 8B). Interestingly, a new spheroid-shaped bone was found in the center of the PCL/nHA + HPCH scaffold (right-sided defect of the PCL/nHA + HPCH group, Fig. 8A).

In keeping with the micro-CT findings, H&E staining and Masson's trichrome staining revealed that the MSC-encapsulated PCL/nHA + HPCH hybrid scaffold had greater amounts of regenerated bone after nine weeks. H&E staining after nine weeks revealed new bone formation in all groups; in the control and PCL/nHA groups, new bone appeared mainly in the defect margins. Some soft tissue was retained within the scaffold (Figs. 8C-a-d). In contrast, in the HPCH group, new bone formed at the margin of the defect and grew into the channel of the hybrid scaffold; noticeable fibrous tissue formation was observed in the defect space, where bone formation was absent (Figs. 8C-f). Interestingly, a new round-shaped bone was found in this group (Fig. S7), consistent with micro-CT results (Fig. 8A).

**Figure 9:**
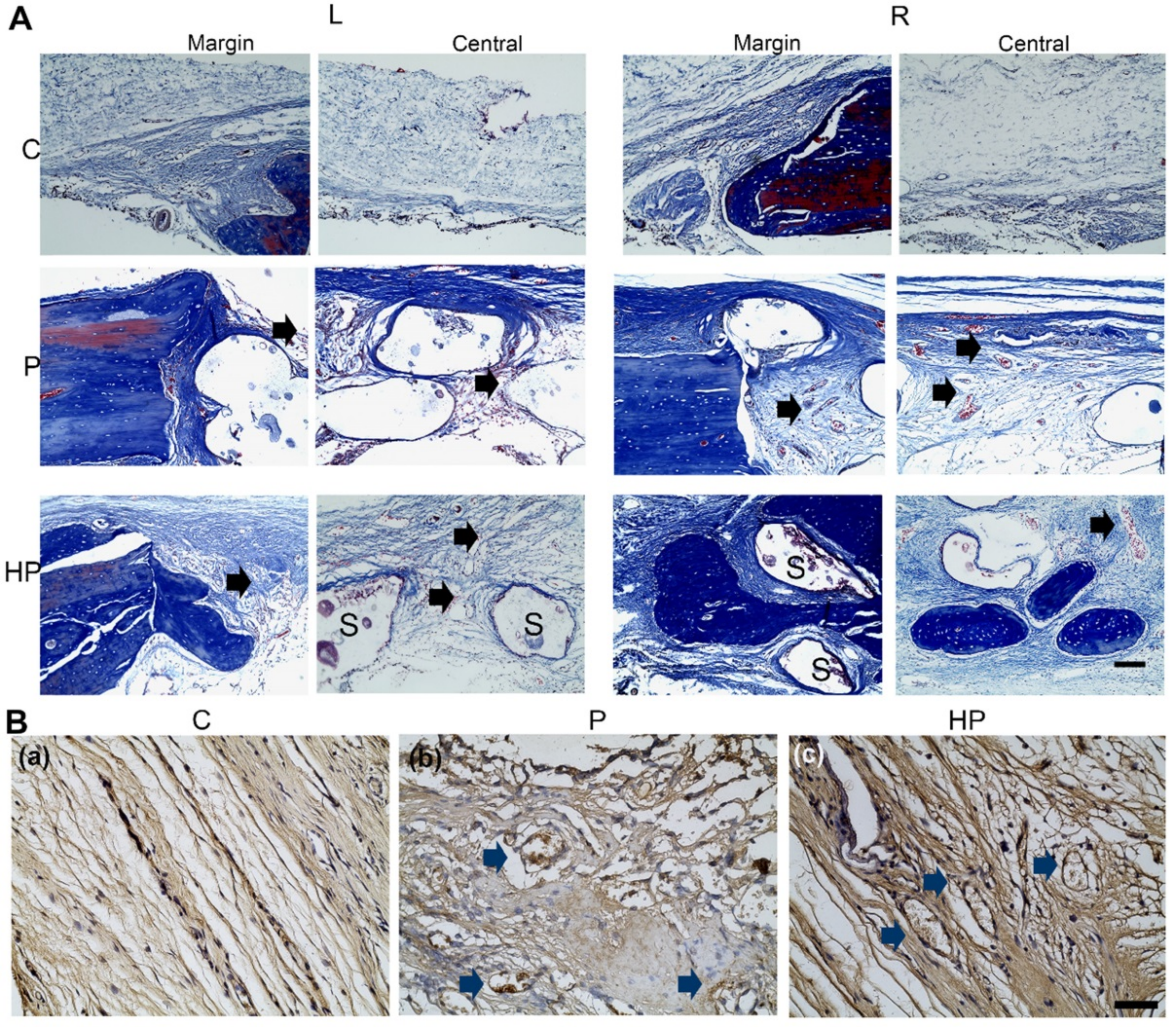
** Masson staining and CD31 immunochemistry staining of calvarial defects at 9-week post-surgery.** (A) Masson staining of newly formed bone in the margin and center of the calvarial defect. Scale bar: 100 μm. L, left defect; R, right defect; NB, new bone; S, scaffold. The black arrows indicated the new vessels within the scaffolds. (B) Immunohistochemical staining for CD31 expression. The blue arrows indicated the CD31 positive staining around the vessel. Scale bar: 50 μm. C, control; P, PCL/nHA; HP, PCL/nHA+HPCH.

The results of Masson's trichrome staining showed that, in the control group, a thin fibrous tissue layer connected the margins of the defect. In contrast, in the PCL/nHA and PCL/nHA + HPCH groups, dense fibrous connective tissue occupied the defects. In the right-sided defect of the PCL/nHA + HPCH group, we observed extensive new bone growth into the scaffold from the margin, and round-shape bone formation in the central scaffold (Fig. 9A).

As an indication of angiogenesis of the hybrid scaffold, immunochemistry staining was used to evaluate CD31 expression, a biomarker of vessel ECs^44^. Abundant CD31-positive cells were observed in the hybrid scaffold group under 400× magnification; however, no vessel formation was evident in the control group (Fig. 9B), consistent with the *in vitro* PCR results.

## Discussion

Implantation for bone regeneration must be safe and efficient. The interaction with host cells should be assessed before application in clinical practice. The overall goal of this study was to determine whether the incorporation of MSCs loaded into HPCH and 3D-printed PCL/nHA could promote bone regeneration, and to elucidate the underlying mechanism. In this work, HPCH showed a high cell viability and low toxicity. The 3D-printed customized PCL/nHA scaffold significantly improved the mechanical properties of the hybrid system and stimulated the macrophages to secrete angiogenetic and osteogenetic growth factors. Both *in vitro* and *in vivo* experiments showed that the MSC-encapsulated HPCH efficiently promoted macrophage transition toward the M2 type. The *in situ* calvarial defect implantation showed that incorporation of cell-encapsulated HPCH significantly enhanced the osteoconductivity and osteoinductivity of the PCL/nHA scaffold. Altogether, these results confirmed that a combination of an MSC-loaded thermosensitive HPCH hydrogel and a PCL/nHA scaffold is a promising approach for bone regeneration.

The hybrid scaffold is fundamental in bone and cartilage tissue engineering^45^, ^46^. The combination of synthetic materials and natural hydrogel takes advantage of both materials, which is important given the potential complexity of target repair sites. Gel-based scaffolds show high biocompatibility, but also show a rapid loss of mass results in their failure to maintain their original shape 47. The use of a synthetic polymer as the inner structure can eliminate the need for chemical or physical modification to improve the mechanical properties of the hydrogel 48. Several studies have investigated this concept. Collagen 49, chitosan 50, gelatin 51, 52, silk fibroin 53 and hyaluronic acid 54 have been explored in terms of their ability to transport cells in hybrid scaffolds. However, the chemical agents used to crosslink the hydrogel are potentially harmful to cells. The gelation process cannot be homogeneous, as the cross-linker is unable to fully permeate into the hydrogel when the scaffold is very large. Our novel thermosensitive HPCH hydrogel that is based on physical crosslinking ensures a homogeneous gelation throughout the scaffold in a cell-friendly manner. Use of a customized PCL/nHA scaffold as the inner structure can significantly improve the mechanical properties and prevent rapid mass loss, without disturbing the cells encapsulated in the hydrogel. The separate fabrication of the composite scaffold makes it more suitable for the manipulation of PCL/nHA, and it allows the exclusion of toxic byproducts.

In our previous work, we identified variations in cell behavior based on whether they were encapsulated in HPCH or cultured on the hydrogel 40. In this work, we observed the same phenomenon in MSCs, i.e., when MSCs were encapsulated by HPCH, the cells retained distinct and round shapes; however, on the surface of the hydrogel, MSCs tended to aggregate and form spheroid cells. Interestingly, under a bone-inducting environment, MSCs encapsulated in HPCH showed more significant OCN, OPN, and Runx2 expression, and the expression levels of Runx1 and Runx3, which are related to chondrogenesis, increased simultaneously. We proposed that MSCs underwent both chondrogenesis and hypertrophy, namely endochondral ossification. It is known that the aggregated state of MSCs benefit chondrogenesis 55, accounting for the inhibition of osteogenesis when MSCs were cultured on the surface of HPCH.

Since bone repair is an inflammatory process, macrophages play an important role in successful bone regeneration. Activated macrophages release growth factors, cytokines, and other bioactive agents to modulate the functions of other cells in the inflammatory milieu 56. The use of biomaterials for the delivery of proteins or cells with immunomodulatory capacity may facilitate successful tissue regeneration 57. IL-4 58, 59, PGE2 60, nutrient elements (e.g., Mg, Sr, Si) 61, and anti-inflammatory molecules and peptides 62, have been developed to help biomaterials modulate the immune response. Shifting the macrophages toward a regeneration-promoting state is vital for early angiogenesis and osteoinduction of the implant 63. It has been found that a number of biomaterial strategies, such as surface chemistry, topography, and biomolecules, can modulate the cross-talk between immune response and osteogenesis 64. Besides, Jie Meng et al. 65 proposed that activation of macrophages into a M1/M2 mixed status can support angiogenesis. Daniel Hachim et al. 59 in their study found that shifts in macrophage phenotype at the biomaterial interface can lead to better osteointegration. It has been proposed that the therapeutic effects of MSCs are dependent on their capacity to support a regenerative niche, rather than their ability to differentiate and be incorporated into the host bone 66. Thus, the immunomodulation of MSCs for macrophage polarization was important for the bone formation.

It has been reported that both M1 and M2 macrophages can enhance vascularization, as they participate in angiogenesis in different ways. M1 macrophages are responsible for the initiation of angiogenesis, by secretion of VEGF; M2 are involved in the later stages of angiogenesis, stabilizing the vasculature, and usually secreting PDGF-BB 67. In our research, the PCL/nHA scaffold did not only improve mechanical properties, but it also stimulated macrophages to secrete angiogenesis growth factors, such as VEGF and PDGF-BB, as well as osteogenesis growth factors, including BMP2, TGF-β1, and PGE2. Our results also confirmed that HPCH promotes VEGF expression, whereas modulation of MSCs promotes PDGF-BB secretion. These growth factors guarantee early angiogenesis and bone formation; the *in vivo* CD31 immunochemistry staining and histology staining confirmed the effect of the PCL/nHA scaffold. Thus, encapsulated MSCs act as an immunoregulator that promotes a timely M1-M2 shift, with HPCH acting as a “protector” of MSCs and an “attractor” of macrophages. Both *in vitro* and *in vivo* experiments showed that HPCH attracted macrophages, and MSCs promoted an M1-M2 shift. A significant difference in bone regeneration between the PCL/nHA and PCL/nHA + HPCH groups with addition of MSCs proved that HPCH can effectively “protect” or “entrap” exogenous MSCs, to improve bone volume at the defect site. The significant difference in bone regeneration in the PCL/nHA + HPCH group with addition of MSCs vs. without the addition of MSCs proves that MSCs are the key element in bone regeneration.

There were some limitations to this study. Firstly, we ended our experiment at nine weeks postoperatively, upon noticing PCL/nHA scaffold degradation and dislocations (Fig. 8C). During the early stage of bone repair, the scaffold needs to keep stable before the bone ingrowth. Thus, the degradation period should extend the bone repair process. It has been reported that the degradation of PCL includes a decrease in molecular weight *in vivo* without deformation for more than two years, after which the PCL gradually breaks into pieces and loses strength 68. Further studies should focus on matching the scaffold degradation and bone growth rates. An expanded follow-up study should be carried out to examine whether the scaffold fully degrades, for full repair of the defect. Despite the advantages of the “dual-hole” animal model mentioned above, the risk of contamination of adjacent materials was high. We retained the periosteum as a biological mechanical sleeve, to prevent migration of the implants away from the defect so as to reduce the contamination risk 69. Future studies should utilize larger, more clinically relevant animal models to examine the bone healing ability of massive hybrid scaffolds.

In conclusion, we developed a cost-effective, customized, and easily manipulated biologically active material for bone regeneration, and explored the underlying mechanism, which is important for clinical applications. HPCH is cheap and has high cell viability, and MSC encapsulation entails the simple mixing of two components under low temperature. The ready-to-use PCL/nHA scaffold can be fabricated on an individual patient basis, as the HPCH/MSC mixture is infused into the printed scaffold just before implantation. Therefore, this hydrogel/cell/scaffold system is safe, effective, and promising for BTE.

## Conclusions

In summary, we developed and characterized a HPCH + MSCs hydrogel with a 3D-printed PCL/nHA scaffold for bone regeneration. The MSCs incorporated into the HPCH hydrogel acted not only as a stem cell source for osteogenesis, but also as a paracrine source to promote macrophage regeneration. Increased M2 macrophage polarization at the injury site was identified as a novel mechanism to promote tissue repair. Moreover, the 3D-printed hybrid scaffold showed superior compressive strength and stimulated macrophages to secrete angiogenic and osteoinductive growth factors, creating a favorable micro-environment for vascularization and osteogenesis. Altogether, these findings demonstrate the potential of an HPCH + MSC-infused 3D-printed PCL/nHA scaffold as a novel therapeutic strategy for promoting tissue regeneration.

## Abbreviations

3D: three-dimensional
ALP: alkaline phosphatase
Arg-1: arginase-1
BMP2: bone morphogenetic protein-2
BTE: bone tissue engineering
CCK8: cell counting Kit-8
CCL22: C-C motif chemokine ligand-22
cDNA: complementary DNA
CLSM: confocal laser scanning microscopy
DAPI: 4', 6-diamidino-2-phenylindole
ECs: endothelium cells
EDTA: ethylenediaminetetraacetic acid
FBS: fetal bovine serum
H&E: hematoxylin and eosin
HA: hydroxyapatite
HPCH: hydroxypropyl chitin hydrogel
IL-1: interleukin-1
LPS: lipopolysaccharide
Micro-CT: microcomputed tomography
MMP9: matrix metalloproteinase-9
MSC: mesenchymal stem cell
nHA: nano-hydroxyapatite
OCN: osteocalcin
PCL: poly(ε-caprolactone)
PDGF-BB: Platelet-derived growth factor-BB
PGE2: prostaglandin E2
PI: propidium iodide
SEM: scanning electron microscopy
TGF-β1: transforming growth factor-beta 1
TNF-α: tumor necrosis factor-alpha
VEGF: vascular endothelial growth factor
VOI: volume of interest
