# Supplementary material for: Mesenchymal stem cell-loaded thermosensitive hydroxypropyl chitin hydrogel combined with a three-dimensional-printed poly(ε-caprolactone) /nano-hydroxyapatite scaffold to repair bone defects via osteogenesis, angiogenesis and immunomodulation
Source: Theranostics. 2020 Jan 1;10(2):725–40. doi: 10.7150/thno.39167 (PMC6929983; doi:10.7150/thno.39167)
Supplement: Supplementary file 1 — Supplementary figures and tables. [file thnov10p0725s1.pdf]

Supporting information

Mesenchymal stem cell-loaded thermosensitive hydroxypropyl chitin hydrogel combined with a three-dimensional-printed poly(ε-caprolactone) /nano-hydroxyapatite scaffold to repair bone defects via osteogenesis, angiogenesis and immunomodulation

Xiongfa Ji[1,2], Yuan Xi[2*], Limin Ma[1*], Bo Bi[3], Hao Zhu[2], Zehua lei[2], Wenbin Liu[2],HongXu Pu[2], Jiawei Jiang[2], Xulin Jiang[3✉], Yu Zhang[1✉],Jun Xiao[2✉]*

1.Department of Orthopedics, Guangdong General Hospital, Guangdong Academy of Medical Sciences, Guangzhou, Guangdong 510080, PR China

2.Department of Orthopaedic Surgery, Tongji Hospital, Tongji Medical College, Huazhong University of Science and Technology, Wuhan, 430030, China

3.Key Laboratory of Biomedical Polymers of Ministry of Education & Department of Chemistry, Wuhan University, Wuhan, 430072, China.

*These authors contributed equally to this work.

✉ Xulin Jiang, Yu Zhang and Jun Xiao are the co-corresponding authors.

Table S1. Primers used in Real-Time PCR for RAW 264.7.

Gene	Forward primer sequence(5'-3')	Reverse primer sequence(5'-3')
**GADPH**	TTCCAGGAGCGAGACCCCACTA	GGGCGGAGATGATGACCCTTTT
**BMP2**	GGGACCCGCTGTCTTCTAGT	TCAACTCAAATTCGCTGAGGAC
**TGF β1**	CCACCTGCAAGACCATCGAC	CTGGCGAGCCTTAGTTTGGAC
**Ptges**	GGATGCGCTGAAACGTGGA	CAGGAATGAGTACACGAAGCC
**VEGFa**	TATTCAGCGGACTCACCAGC	AACCAACCTCCTCAAACCGT
**PDGFB**	CATCCGCTCCTTTGATGATCTT	GTGCTCGGGTCATGTTCAAGT
**MMP9**	GCAGAGGCATACTTGTACCG	TGATGTTATGATGGTCCCACTTG
**IL1β**	CCCAACTGGTACATCAGCACCTC	GACACGGATTCCATGGTGAAGTC
**TNF-α**	GGACTAGCCAGGAGGGAGAA	CGCGGATCATGCTTTCTGTG
**IL6**	CTGCAAGAGACTTCCATCCAG	AGTGGTATAGACAGGTCTGTTGG
**Arg-1**	CTCCAAGCCAAAGTCCTTAGAG	GGAGCTGTCATTAGGGACATCA
**IL10**	GCTCTTACTGACTGGCATGAG	CGCAGCTCTAGGAGCATGTG
**CCL22**	CTCTGCCATCACGTTTAGTGAA	GACGGTTATCAAAACAACGCC

Table S2. Primers used in Real-Time PCR for MSCs.

Gene	Forward primer sequence(5'-3')	Reverse primer sequence(5'-3')
**GADPH**	GGTGGACCTCATGGCCTACA	CTCTCTTGCTCTCAGTATCCTTGCT
**OCN**	GCCCTGACTGCATTCTGCCTCT	TCACCACCTTACTGCCCTCCTG
**OPN**	CCAGCCAAGGACCAACTACA	GCTGGCAGTGAAGGACTCAT
**ALP**	GCACAACATCAAGGACATCG	TCAGTTCTGTTCTTGGGGTACAT
**Runx 1**	TGCGTATCCCCGTAGATGCC	GTGGTCAGCTAGTACCTCCAC
**Runx 2**	GGGACCGACACAGCCATATA	TCTTAGGGTCTCGGAGGGAA
**Runx 3**	TGTAACACCAAGCACACCCA	GTTCAGGTCTGAGGAGCCTTG
**COL I**	GGCAGATGGTGGATGGTAAGT	CCAGACTCTTCAAAACCTCGCT
**COL X**	TGCTGCTAGTGTCCTTGACG	CCTCTTACTGAAATCTCTTTACCCT

Fig.S1 The SEM characterization of nano-hydroapatite(nHA). Scale bar, 200μm.

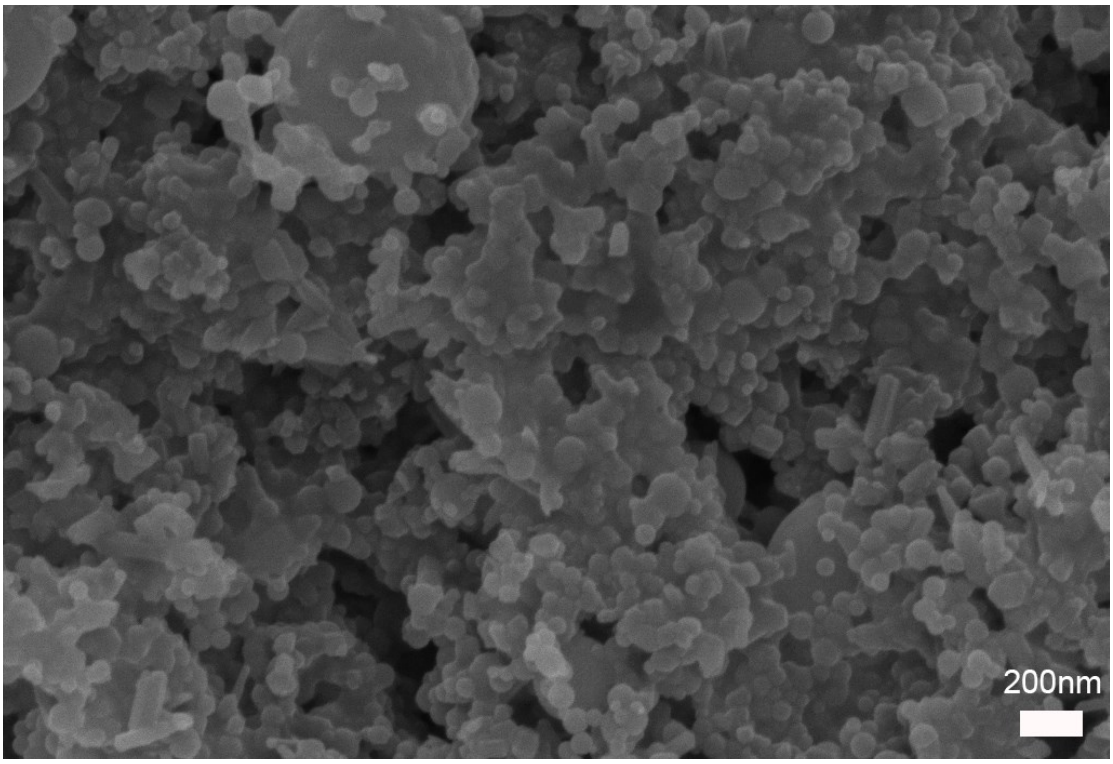

Fig.S2 The identification of rat endothelial cells using immune-fluorescent staining of CD31. Scale bar, 50μm.

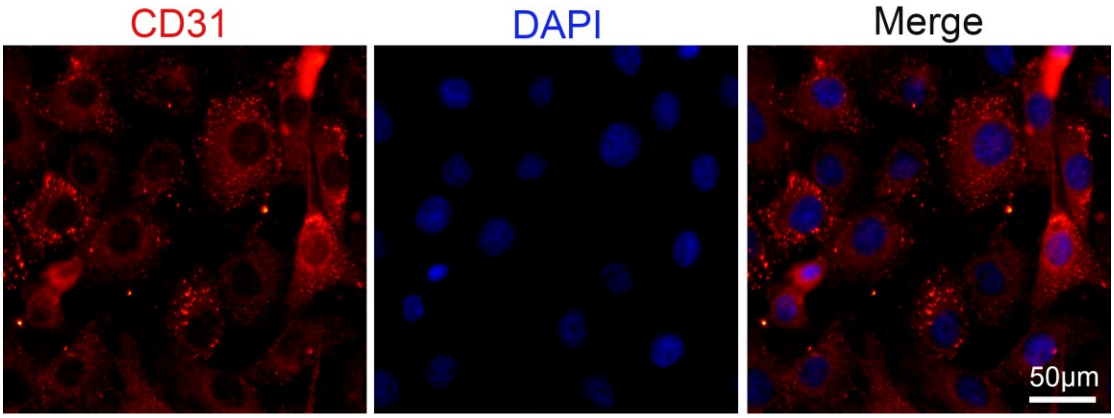

Fig.S3 MSC spheroids formation in or on HPCH. MSCs encapsulated in (A) HPCH hydrogel and on (B) HPCH hydrogel after 1 day, 4 days and 7 days' culture.

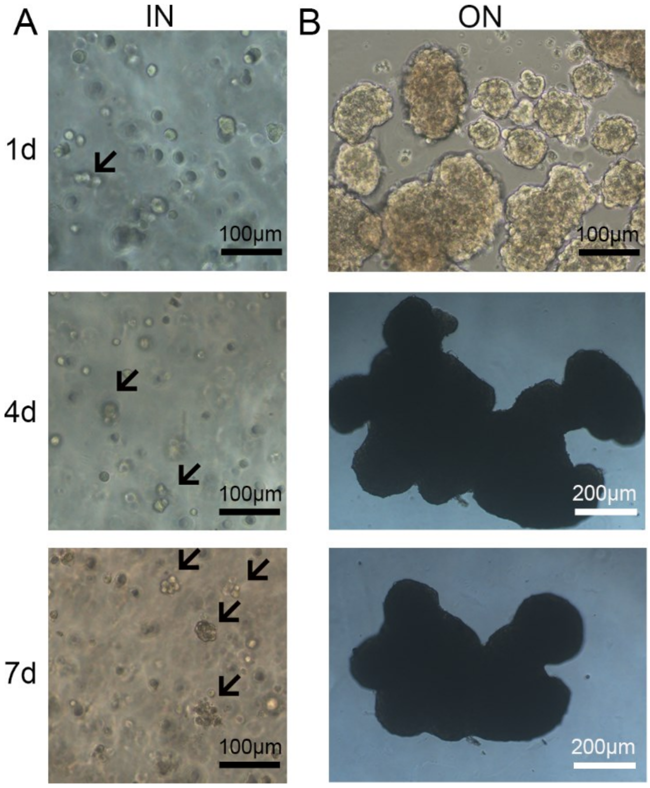

Fig. S4 Alizarin red staining of MSCs for 14 days' culture. The culture medium was the extract medium from RAW 264.7 cultured with PCL/nHA or HPCH. Scale bar, 1mm.

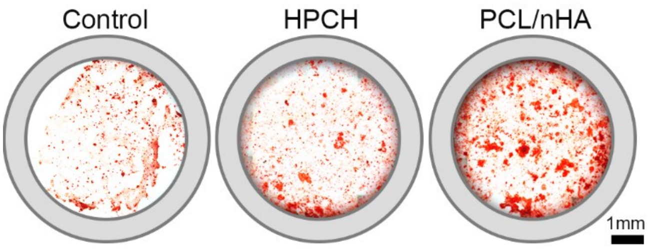

Fig.S5 Immunofluorescent staining of the subcutaneous injection of MSCs. Scale bar, 100 μm.

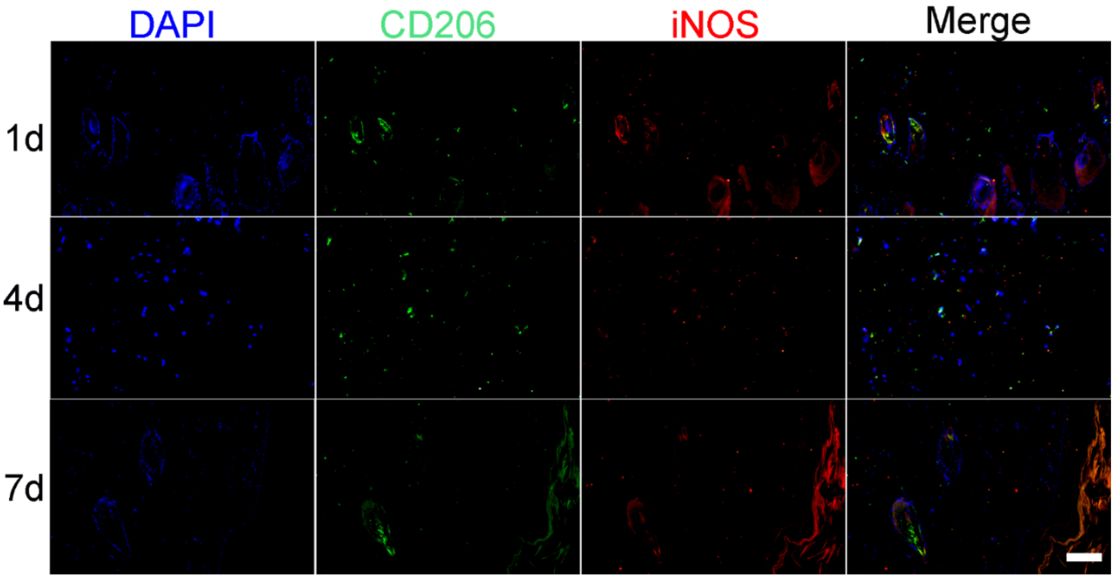

Fig. S6 H&E staining for the subcutaneous implantation of MSCs and HPCH for 1d, 4d and 7d. Scale bar, 400 μm.

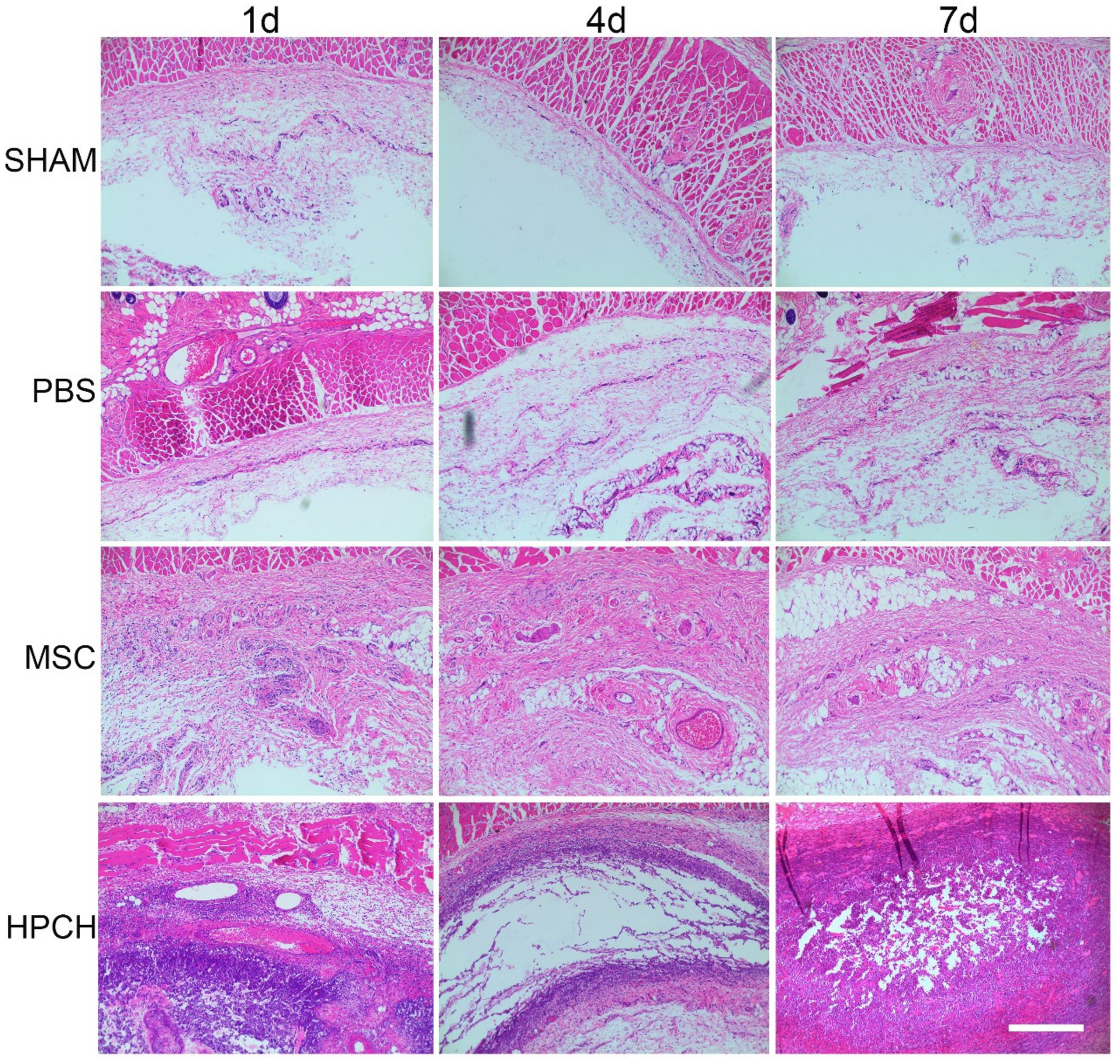

Fig.S7 New bone formed in the central of the scaffold. Concentrically arranged lamellae of bone matrix and osteocytes lived within lacuna, and marrow cavity was formed in the “osteon”. S, scaffold. Scale bar, 100μm.

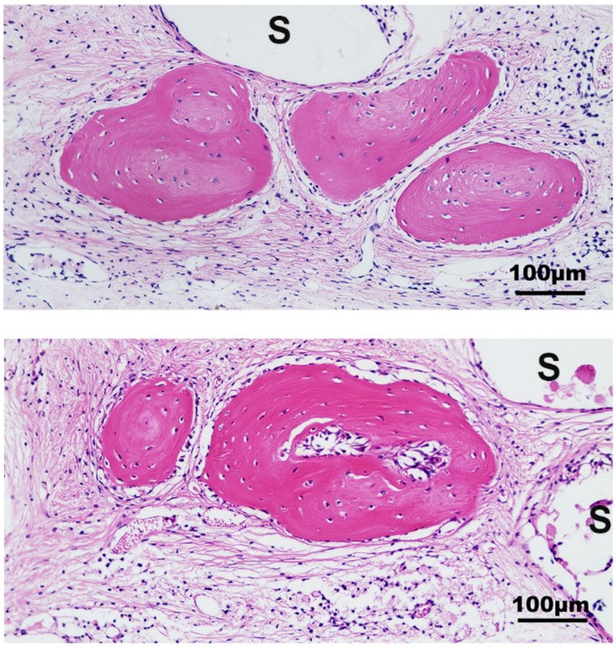

Video 1. 3D reconstruction of PCL/nHA based on micro-CT result.
